# Mast cell stabilizer disodium cromoglycate improves long-term cognitive impairment after general anesthesia exposure in neonatal mice

**DOI:** 10.3389/fnins.2022.990333

**Published:** 2022-09-15

**Authors:** Xiaojun Zhang, Wensi Wu, Zhenzhen Zheng, Liang Li, Junjun Chen, Junying Zhong, Le Zhao, Jiawei Chen, Zhi Wang, Fanqing Meng

**Affiliations:** ^1^Department of Anesthesiology, Sun Yat-sen Memorial Hospital, Sun Yat-sen University, Guangzhou, Guangdong, China; ^2^Department of Thoracic Surgery, Qilu Hospital of Shandong University, Jinan, Shandong, China; ^3^Department of Anesthesiology, Qilu Hospital of Shandong University, Jinan, Shandong, China; ^4^Department of Anesthesiology, Jinan Maternity and Child Care Hospital, Jinan, Shandong, China

**Keywords:** mast cell, disodium cromoglycate, long-term cognitive impairment, general anesthesia, neonatal mice

## Abstract

**Background:**

Prolonged exposure to general anesthesia (GA) results in long-lasting cognitive impairment, especially during critical stages of brain development. An exaggerated neuroinflammation induced by anesthesia is generally considered to be a key cause of cognitive impairment.

**Materials and methods:**

Postnatal day 7 (PND 7) mice were exposed to GA by isoflurane inhalation for 6 h or mock anesthesia. Disodium cromoglycate (DSCG) was intraperitoneally injected daily for 2 weeks, beginning from 30 min before anesthesia. The post-anesthesia evaluation included behavioral tests, toluidine blue staining, immunofluorescence and western blot.

**Results:**

Our results demonstrated the long-term cognition were impaired after 6 h GA exposure in neonatal mice. DSCG treatment ameliorated early mast cells (MCs) degranulation and mast cell tryptase (MCT) expression, which helps to attenuate subsequent neuroinflammation, activation of microglia and astrocytes, and damage to oligodendrocytes and synapses to improve cognitive impairment.

**Conclusion:**

Disodium cromoglycate could effectively improve long-term cognitive impairment after GA exposure in neonatal mice.

## Introduction

Long exposure to GA in neonatal rodents during critical stages of brain development is closely associated with memory and learning impairment, neuroinflammation and brain cell apoptosis (Vutskits and Xie, 2016; Diana et al., 2020). This problem begs the question of how GA can be used more safely in the very young age. One possibility would be co-administrated of protective agents to minimize the determined effects of GA.

The possibility that exposure to GA induces neuroinflammation in the developing brain has also been explored (Vutskits and Xie, 2016; Baud and Saint-Faust, 2019; Liu et al., 2021). Microglia, the primary resident immune cells in the brain, participating protective immune responses, and promoting invasive inflammation in central nervous system disorders (Woodburn et al., 2021; Wu J. et al., 2021). Anesthesia/surgery could induce microglial activation and release inflammatory factors, such as interleukin (IL)-1β, land tumor necrosis factor-alpha (TNF-α) (Vutskits and Xie, 2016; Baud and Saint-Faust, 2019). Overactivated microglia and neuroinflammation cause a neurotoxic response and induce synaptic impairment, resulting in cognitive dysfunction (Liddelow et al., 2017).

Previous studies found that MCs are considered as first responders, are able to initiate and further magnify immune responses in the brain (Zhang et al., 2016b; Hendriksen et al., 2017; Skaper et al., 2017). Mast cells could rapidly respond to inflammatory stimuli and communicate with nearby cells *via* a process called degranulation, further stimulating microglia activation (Zhang et al., 2016a; Hu et al., 2021). Regulation of MCs might be an important means of treating brain disease. Disodium cromoglycate, as mast cell stabilizer, could limit microglial activation by inhibiting mast cell degranulation (Wang et al., 2021). To understand the process, we hypothesized that MCs might initiate the neuroinflammation caused by long anesthesia exposure in postnatal mice, and inducing the subsequent changes of microglia, astrocyte, oligodendrocyte and synapse, and we investigated the role of DSCG on the process.

Given this background, we hypothesized that brain MCs, the first responders in the brain after postnatal long GA exposure, are responsible for inducing neuroinflammation, dysregulating glial cells and impairing synapse, starting a vicious cycle and causing cognitive dysfunction.

## Materials and methods

### Animals

This study was approved by the animal care committee of Sun Yat-sen University and performed in accordance with the National Institutes of Health Guidelines for the Use of Laboratory Animals. C57BL/6 pregnant mice (Sun Yat-sen University, Guangzhou, China, permission number: SCXK 2021–0029) were housed in specific pathogen free environment kept at 22°C and 40–60% humidity with a 12 h light-dark cycle (light from 07:00 to 19:00). Offspring, delivered spontaneously, began experiments at Postnatal day 7 (PND 7) and litter size was kept in around five pups/litter to minimize weight difference among neonatal mice.

### Experimental protocol

**FIGURE 1:**
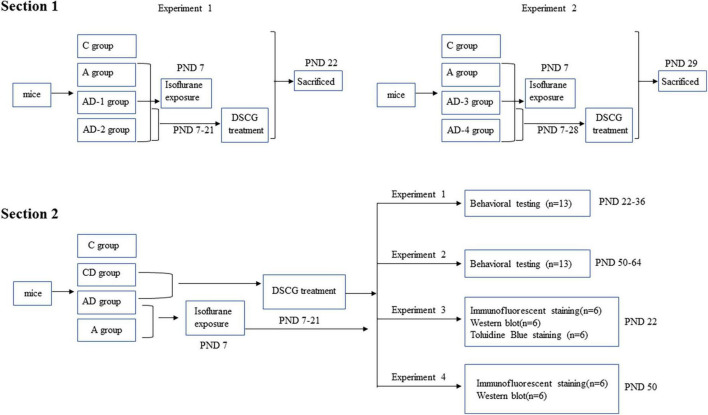
Diagram of timeline of experimental procedures.

As shown in Figure 1, this study was divided into two separate sections.

In section 1, we mainly evaluated the effect of different doses and durations of DSCG on MCT expression to formulate the subsequent experimental protocol. Two independent experiments were performed in this section.

In experiment 1, mice (PND 7) from each litter (4 L, 6 mice pups in each litter, 24 mice pups in total), regardless of gender, were randomly allocated into one of four treatment protocols: sham controls (C), GA-treated (A), GA + DSCG treated group 1 (AD-1), GA + DSCG treated group 2 (AD-2).

In experiment 2, neonatal mice with the same condition were randomly allocated into another one of four treatment protocols: sham controls (C), GA-treated (A), GA + DSCG treated group 3 (AD-3), GA + DSCG treated group 4 (AD-4).

Mice in C group did not receive any intervention; mice in A group received only isoflurane exposure on PND 7; mice in AD-1 group received isoflurane exposure on PND 7 and DSCG intraperitoneally injection during PND 7–21 (25 mg/kg/day, 2 Weeks); mice in AD-2 group received isoflurane exposure on PND 7 and DSCG intraperitoneally injection during PND 7–21 (50 mg/kg/day, 2 Weeks); mice in AD-3 group received isoflurane exposure on PND 7 and DSCG intraperitoneally injection during PND 7–28 (25 mg/kg/day, 3 Weeks); mice in AD-4 group received isoflurane exposure on PND 7 and DSCG intraperitoneally injection during PND 7–28 (50 mg/kg/day, 3 Weeks). Twenty-four hours after the DSCG treatment phase, brain tissues were harvested from each group to detect the expression levels of MCT (PND 22 and PND 29, respectively).

In section 2, we mainly verified the pharmacological effect of DSCG. Mice (PND 7) from each litter (40 L, 5–8 mice pups in each litter, 224 mice pups in total), regardless of gender, were randomly allocated into one of four treatment protocols: sham controls (C), sham + DSCG treated (CD), GA-treated (A), GA + DSCG treated (AD).

Mice in C group did not receive any intervention; mice in CD group received only DSCG intraperitoneally injection during PND 7–21 (25 mg/kg/day, 2 Weeks); mice in A group received only isoflurane exposure on PND 7; mice in AD group received isoflurane exposure on PND 7 and DSCG intraperitoneally injection during PND 7–21 (25 mg/kg/day, 2 Weeks). After that, four independent experiments were performed in this section.

In experiment 1, behavioral testing was performed during PND 22–36 (4 Weeks) in each group.

In experiment 2, behavioral testing was performed during PND 50–64 (8 Weeks) in each group.

In experiment 3, mice were sacrificed at PND 22 (4 Weeks). Brain was harvested to measure MCs by toluidine blue staining, determine the expression of MCT, chondroitin sulfate proteoglycan 4 (CSPG4), oligodendrocyte transcription factor 2 (OLIG2), myelin basic protein (MBP), synaptosomal associated protein 25 kDa (SNAP25), postsynaptic density protein 95 kDa (PSD95) and brain-derived neurotrophic factor (BDNF) by Western blotting, and detect glial fibrillary acidic protein (GFAP) and ionized calcium binding adapter molecule 1 (IBA1) by Immunofluorescent staining.

In experiment 4, mice were sacrificed at PND 50 (8 Weeks). Brain was harvested to determine the expression of MCT, CSPG4, OLIG2, MBP, SNAP25, PSD95, and BDNF by Western blotting, and detect GFAP and IBA1 by Immunofluorescent staining.

Mice did not receive any intervention during PND 22–49 in experiment 2 and 4.

### General anesthesia exposure

In this study, isoflurane (R510-22, RWD, China) was used as the agent for GA exposure. Isoflurane was administrated in a step-down protocol over 6 h (2% Iso hrs. 0–2; 1.4% Iso hrs. 2–4; 0.8% Iso hrs. 4–6) using a transparent anesthesia induction chamber with CO_2_ absorbent pellets to perform GA (Chinn et al., 2019). Fresh gas was 0.5 L/min, and FiO_2_ was about 0.5. The volatile anesthetic concentration was monitored with anesthesia monitor (B450, GE, United States), and the heating blanket (69020, RWD, China) used to maintain rectal temperature of the mice at 37°C. Besides, skin temperature was measured by an infrared thermometer every 10 min. After isoflurane exposure, the chamber was flushed with atmospheric air and mice were allowed to emerge from anesthesia after return of righting reflex, and the pups were returned to their respective dam to continue housing.

### Drug administration

The dose (25 and 50 mg/kg/day, intraperitoneally injection) and durations (2 and 3 Weeks) of DSCG (D129313, Aladdin, China) was chosen according to previous studies in mice (Sun et al., 2007; Furukawa et al., 2020; Li et al., 2021). We initiated a daily treatment with DSCG 30 min before GA. The dose used in this study is below the dose used in humans according to surface area conversion. Disodium cromoglycate was prepared freshly each day by dissolving the powder in phosphate buffer saline (PBS) (C10010500BT, Gibco, United States).

### Behavioral testing

In experiments 1 and 2 of the second section, locomotor activity and postoperative anxiety were detected by open field test, learning and memory were evaluated by novel object recognition (NOR) and Barnes maze test. All behavioral tests were conducted at 10:00 a.m.–5:00 p.m. in a sound-isolated room. And these mice were not subjected to biochemical tests to avoid the possible effects of behavioral tests.

### Open field

Similar to our previous experiments (Zhang et al., 2021), to evaluate the difference between locomotor activity and anxiety, during the open field test, mice moved freely for 5 min in an open-field apparatus consisting of a black opaque plastic chamber (60 cm × 60 cm × 50 cm, ZH-OFT, Anhui Zhenghua Biological Instrument Equipment Co., Ltd., Anhui, China). The behaviors of mice were recorded and analyzed by a video tracking system (Smart v3.0.06, Panlab Harvard Apparatus, Barcelona, Spain). The field was cleaned with 75% ethanol after each test.

### Novel object recognition

To evaluate the mice short-term and long-term memory as previous studies (Lai et al., 2021). One hour after open field test, mice were put into another chamber for habituation for 5 min to perform NOR on the learning phase, two identical objects were placed at adjacent angles of the chamber. Mice were put into the chamber with their backs toward the objects and allowed to explore the chamber freely for 5 min. At this stage, the mice would be excluded if the total exploration time on two objects was less than 5 s. On the testing phase, one of the objects was replaced by a novel object 30 s or 24 h later. Mice explored freely for 5 min in the same way as above. The mice behavior was recorded and analyzed by video tracking system (Smart v3.0.06, Panlab Harvard Apparatus, Barcelona, Spain). Exploration time of old (T1) and new (T2) objects in 5 min was recorded, and the memorization ability of the mice was quantified by discrimination index (DI) = T2/ (T1 + T2). The DIs at 30 s and 24 h on testing phase reflected the instant and long-term memory, respectively. The objects and field were cleaned with 75% ethanol after each test.

### Barnes maze

Twenty-four hours after NOR test, mice were subjected to Barnes maze to test their short-term and long-term spatial learning and memory as previous studies (Wu W. et al., 2021). Barnes maze is a circular platform with 20 equally spaced holes (Anhui Zhenghua Biological Instrument equipments Co., Ltd., Anhui, China). One of the holes was connected to a dark chamber that was called target box. Aversive noise (85 dB) and bright light (200 W) shed on the platform were used to encourage mice to find the target box. The mice had a spatial acquisition phase that lasted for 4 days with 4 min per trial, 2 trials per day and 15 min between each trial. After training for 4 days, their spatial learning and memory was tested on day 5 (short-term) and day 12 (long-term). No test or handling was performed from day 5 to day 12. The latency to enter the target box during each trial was recorded and analyzed by video tracking system (Smart v3.0.06, Panlab Harvard Apparatus, Barcelona, Spain). The maze was cleaned with 75% ethanol after each test.

### Toluidine blue staining of brain section

For determination of MCs, brain tissues were fixed, dehydrated, embedded and cut into 20 μm-thick coronal section. Brain sections were stained with toluidine blue (G3668, Solarbio, China) according to standard protocols. The MCs were determined microscopically in 10 systematically selected brain sections containing hippocampal cornus ammonis 1 (CA1) region. All procedure were carried out on coded slides by a single observer.

### Western blot

Protein concentrations of samples were determined by bicinchoninic acid protein assay (Beyotime, P0010S, China). Ten to thirty micrograms of each sample were subjected to Western blot using the following primary antibodies: rabbit monoclonal anti-MCT (Beyotime, AF2059, China) at 1:1,000 dilution, rabbit polyclonal anti-CSPG4 (Beyotime, AF5144, China) at 1:1,000 dilution, rabbit monoclonal anti-OLIG2 (Beyotime, AF1312, China) at 1:1000 dilution, rabbit polyclonal anti-MBP (Boster, BA0094, China) at 1:1,000 dilution, rabbit polyclonal anti-SNAP25 (Beyotime, AF8016, China) at 1:1,000 dilution, rabbit monoclonal anti-PSD95 (Beyotime, AF1096, China) at 1:1,000 dilution, rabbit monoclonal anti-BDNF (Beyotime, AF1423, China) at 1:1,000 dilution, rabbit monoclonal anti-β-tubulin (Beyotime, AF1216, China) at 1:1,000 dilution. The secondary antibodies used were horseradish peroxidase (HRP)–conjugated goat anti-rabbit lgG (1:2,000, A0208, Beyotime, China). Images were scanned by Chemiluminescent Imaging System (SmartChemi 910 plus, Sega, China) and analyzed using Image J (National Institutes of Health, Bethesda, MD, United States). The band signals of interested proteins were normalized to those of the corresponding β-tubulin and expressed as folds of control sample from the same gels.

### Immunofluorescent staining

Immunofluorescent labeling was performed as previous studies to detect GFAP and IBA1 in hippocampal CA1 region (Lai et al., 2021). Briefly, mice were sacrificed under deep anesthesia and transcardially perfused with PBS and 4% paraformaldehyde (PFA) (SJ-BL539A, Biosharp, China). Brains were harvested and fixed in 4% PFA at 4°C for 24 h, dehydrated, and embedded in optimal cutting temperature compound. Coronal 20-μm thick sections were cut and mounted on microscope slides. After being washed in PBS, sections were blocked in 5% goat serum (AR0009, Boster, China) in PBS containing 0.3% Triton-X 100 (ST795, Beyotime, China) for 1 h at room temperature (RT) and then incubated at 4°C overnight with primary antibodies: mouse monoclonal anti-GFAP antibody (1:50, Beyotime, AF0156, China) and rabbit monoclonal anti-IBA1 antibody (1:100, ab178847, United States). Sections were rinsed in PBS with 0.3% Triton-X 100. The goat anti-mouse IgG antibody conjugated fluorescein isothiocyanate (1:500, Beyotime, A0568, China) and goat anti-rabbit IgG antibody conjugated cyanine 3 (1:500, Beyotime, A0516, China) were incubated with sections for 2 h at RT in the dark. The sections were washed in PBS, incubated with 4’,6-diamidino-2’-phenylindole (Servicebio, G1012, China) for nuclear staining, rinsed and mounted with antifade mounting medium (Beyotime, P0128M, China). Images of immunostaining were acquired by LSM 800 microscope system (LSM 800 with aiyscan, Zeiss, Germany), and a negative control omitting the incubation with the primary antibody was included in all experiments. The positive stained area for one interested marker was quantified using the Image J (National Institutes of Health, Bethesda, MD, United States), and presented as ratio of average fluorescence intensity and fluorescence area. All quantitative analyses were performed in a blinded manner.

### Statistical analysis

All data that were collected were included in analyses. The number of animals that contributed data for analysis in each experimental condition was stated in figure legends. Results were presented as means ± S.E.M. in normal distribution data. Data from the training sessions of Barnes Maze were analyzed by one-way (for comparisons within a group) repeated analysis of variance (ANOVA) followed by Tukey’s test. The other data were analyzed by one-way ANOVA followed by Tukey’s test with normally distributed data. Differences were considered significant at a *p* < 0.05. All statistical analyses were performed with GraphPad Prism 8.0 (San Diego, CA, United States).

## Results

### Disodium cromoglycate reduced the expression of mast cell tryptase after early general anesthesia exposure

**FIGURE 2:**
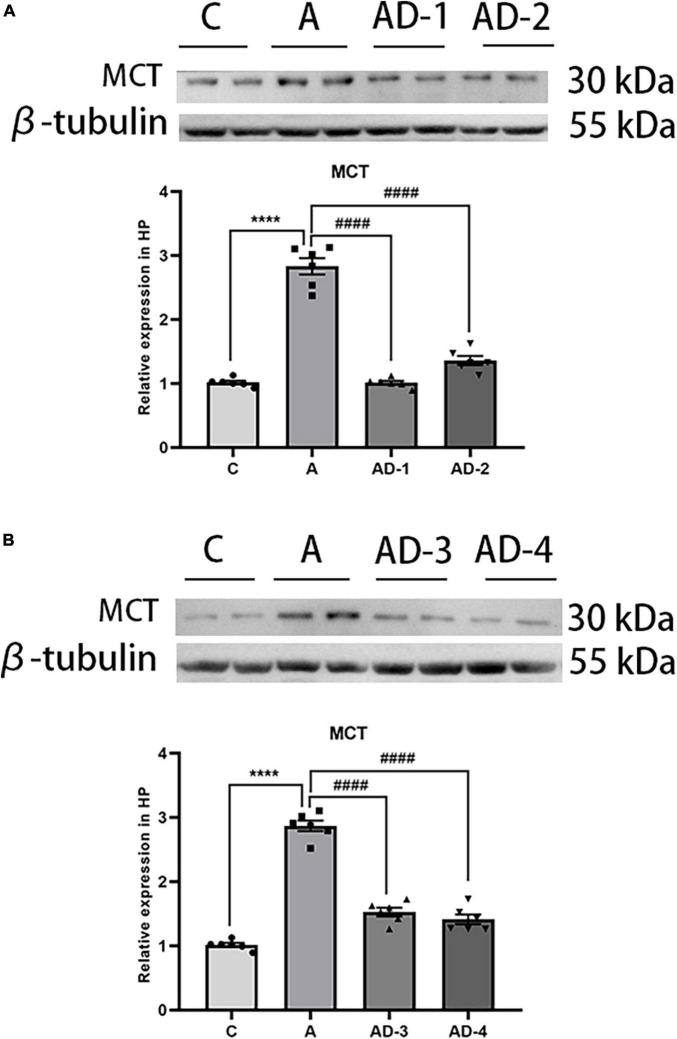
Effects of different treatment doses and durations of Disodium cromoglycate (DSCG) on mast cell tryptase (MCT) expression in hippocampus. **(A)** Representative western blot images and graphic presentation of MCT protein abundance after 2 weeks DSCG treatment. **(B)** Representative western blot images and graphic presentation of MCT protein abundance after 3 weeks DSCG treatment. Results are median ± S.E.M (*n* = 6) with the presence of individual animal data in the bar graphs. Results were analyzed by one-way analysis of variance (ANOVA) followed by the Tukey’s test. ****P < 0.0001 compared with C group. ^####^*P* < 0.0001 compared with A group.

As a marker of MCs and degranulation, we first detected the effect of early GA exposure and (or) DSCG on MCT expression. As shown in Figure 2, the results suggested that the expression of MCT was significantly increased after early GA exposure, and DSCG treatment effectively reduced the expression of MCT. In addition, we found that different doses (25 and 50 mg/kg/day) and different durations (2 and 3 Weeks) of DSCG did not significantly affect the expression of MCT after early GA exposure. Referring to previous studies (Sun et al., 2007; Furukawa et al., 2020), in the follow-up experiments in this study, we treated mice with a dose of 25 mg/kg/day for 2 weeks to avoid potential side effects.

### Disodium cromoglycate ameliorated long-term cognitive impairment after early general anesthesia exposure

**FIGURE 3:**
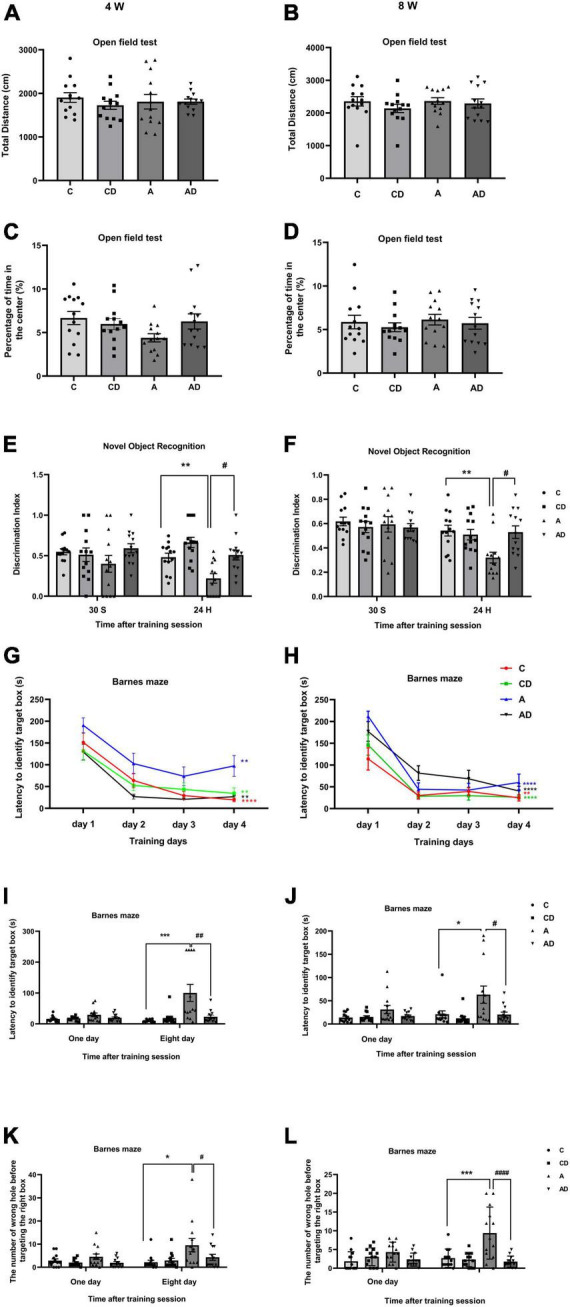
Behavioral performances of different groups of mice in adolescence and adulthood. **(A)** Total distance in the open field test at 4 Weeks. **(B)** Total distance in the open field test at 8 Weeks. **(C)** The percentage of time in center in the open field test at 4 Weeks. **(D)** The percentage of time in center in the open field test at 8 Weeks. **(E)** Performance in the novel object recognition (NOR) test at 4 Weeks. **(F)** Performance in the NOR test at 8 Weeks. **(G)** Performance in the training session of Barnes maze at 4 Weeks. **(H)** Performance in the training session of Barnes maze at 8 Weeks. **(I)** Latency to identify target box in the test session of Barnes maze at 4 Weeks. **(J)** Latency to identify target box in the test session of Barnes maze at 8 Weeks. **(K)** The number of wrong holes explored in the test session of Barnes maze test at 4 Weeks. **(L)** The number of wrong holes explored in the test session of Barnes maze test at 8 Weeks. Results are median ± S.E.M (*n* = 13) with the presence of individual animal data in the bar graphs. Results were analyzed by one-way analysis of variance (ANOVA) followed by the Tukey’s test. *P < 0.05, **P < 0.01, ***P < 0.001 compared with C group. ^#^*P* < 0.05, ^##^*P* < 0.01, ^####^*P* < 0.0001 compared with A group. In Barnes maze training phase, **P < 0.01 for the comparisons of values of day 4 with those of day 1. ****P < 0.0001 for the comparisons of values of day 4 with those of day 1.

Based on the identified dose (25 mg/kg/day) and durations (2 Weeks), we tested the effect of DSCG on early GA exposure-induced long-term cognitive impairment in neonatal mice. Mice were assessed by open field, NOR and Barnes maze tests at two phases, 4 weeks (represent adolescence) and 8 weeks (represent early adulthood) old (Boscolo et al., 2013; Van Hees et al., 2022). In the open field test, the results showed early GA exposure and (or) DSCG had no effect on locomotor activity or anxiety behavior in all groups (Figures 3A–D). In the NOR test, DSCG improved long-term cognitive impairment after early GA exposure in both ages of mice (Figures 3E,F). Mice in all groups took less time to find the target box after the Barnes maze training session (Figures 3G,H), indicating the development of performance after training. The results of test phase of Barnes maze (Figures 3I–L) and NOR test in both ages indicating early GA exposure impaired long-term cognition in mice from adolescence to early adulthood. Disodium cromoglycate treatment offered a protection of long-term cognition that lasting into early adulthood. Besides, no negative effects of DSCG on normal mice were found in behavioral tests.

### Disodium cromoglycate inhibited early general anesthesia exposure-induced neuroinflammation associated with mast cells degranulation

**FIGURE 4:**
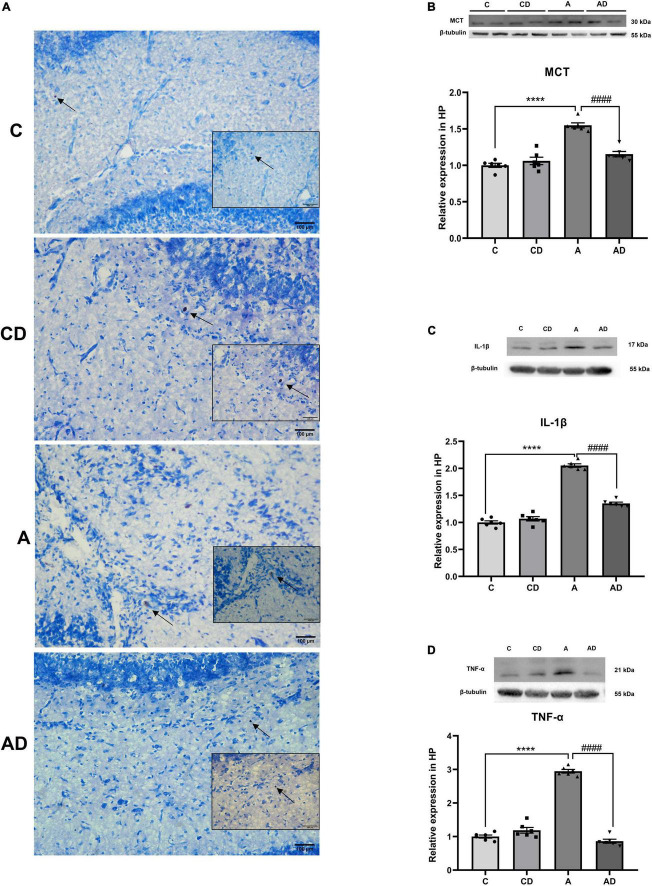
Effects of Disodium cromoglycate (DSCG) and general anesthesia (GA) on mast cells (MCs) degranulation and neuroinflammation in hippocampus. **(A)** Representative images of MCs in hippocampal CA1 region. Black arrows represent enlarged MCs. **(B)** Representative western blot images and graphic presentation of mast cell tryptase (MCT) protein abundance. **(C)** Representative western blot images and graphic presentation of interleukin (IL)-1β protein abundance. **(D)** Representative western blot images and graphic presentation of tumor necrosis factor-alpha (TNF-α) protein abundance. Results are median ± S.E.M (*n* = 6) with the presence of individual animal data in the bar graphs. Results were analyzed by one-way analysis of variance (ANOVA) followed by the Tukey’s test. ****P < 0.0001 compared with C group. ^####^*P* < 0.0001 compared with A group.

To further verify whether MCs degranulation are involved in early GA exposure-induced long-term cognitive impairment, we stained brain hippocampal slices with toluidine blue form each group. As shown in Figure 4, degranulation of MCs and the expression of MCT were significantly induced in early GA-exposed mice. Disodium cromoglycate effectively stabilized MCs degranulation and deceased the expression of MCT (Figures 4A,B). Long-term cognitive impairment after early GA exposure is associated with neuroinflammation, MCs in the brain can respond early to inflammatory stimuli. We further explored whether changes in MCs associated with neuroinflammation. The results exhibited that early GA exposure significantly increased of the expression of TNF-α and IL-1β in hippocampus, and the effect was reversed by DSCG (Figures 4C,D).

### Disodium cromoglycate inhibited early general anesthesia exposure-induced overactivation of microglia and astrocyte

**FIGURE 5:**
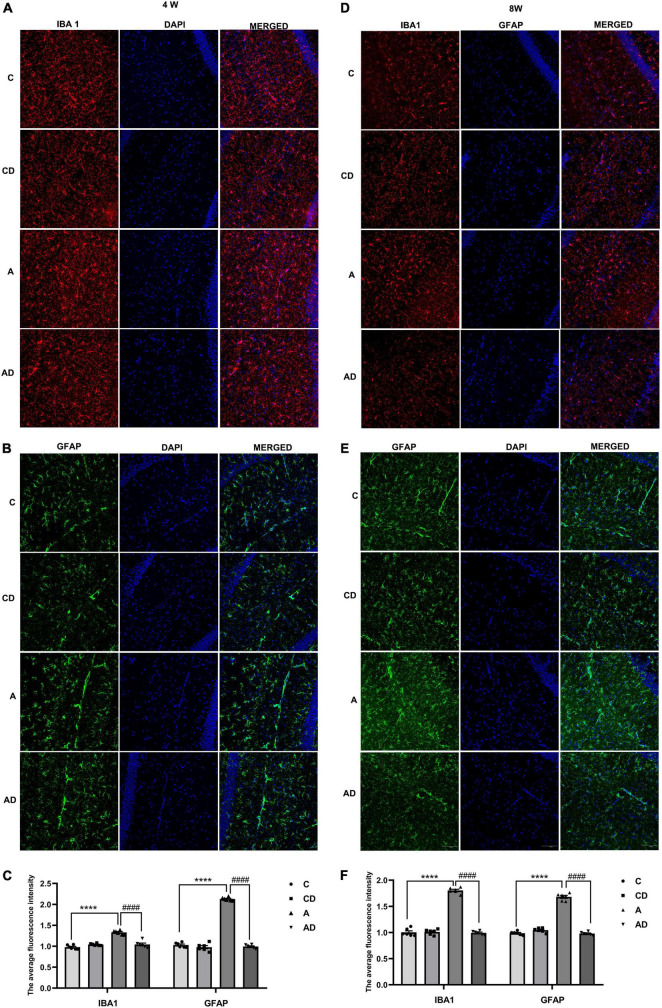
Effects of Disodium cromoglycate (DSCG) and general anesthesia (GA) on the activation of microglia and astrocyte in hippocampal CA1 region. **(A)** Representative ionized calcium binding adapter molecule (IBA1) immunostaining images in 4 Weeks. **(B)** Representative glial fibrillary acidic protein (GFAP) immunostaining images in 4 Weeks. **(C)** Quantitative data of ionized calcium binding adapter molecule 1 (IBA1) and GFAP immunostaining in 4 Weeks. **(D)** Representative IBA1 immunostaining images in 8 Weeks. **(E)** Representative GFAP immunostaining images in 8 Weeks. **(F)** Quantitative data of IBA1 and GFAP immunostaining in 8 Weeks. Scale bar = 100 μm. Results are median ± S.E.M (*n* = 6) with the presence of individual animal data in the bar graphs. Results were analyzed by one-way analysis of variance (ANOVA) followed by the Tukey’s test. ****P < 0.0001 compared with C group. ^####^*P* < 0.0001 compared with A group.

After MCs degranulation, the further increase of TNF-α and IL-1β was associated with the overactivation of microglia and astrocytes. In order to explore the effects of DSCG on microglia and astrocyte, immunofluorescent staining was used to detect IBA1 and GFAP in hippocampal CA1 region, the marker for microglia and astrocyte, respectively. Early GA exposure led to notable increases in microglia activation. As shown in Figure 5, activated microglia had greater cell body, poorly ramified short and thick processes. The effect could be improved through treatment with the DSCG (Figures 5A,C,D,F). We further observe the effect of MCs on astrocyte. The results showed early GA exposure induced increases in activation of astrocyte in hippocampal CA1 region, and DSCG could reverse the status of astrocyte (Figures 5B,C,E,F).

### Disodium cromoglycate ameliorated early general anesthesia exposure-induced dysregulation of oligodendrocyte and synaptic related proteins

**FIGURE 6:**
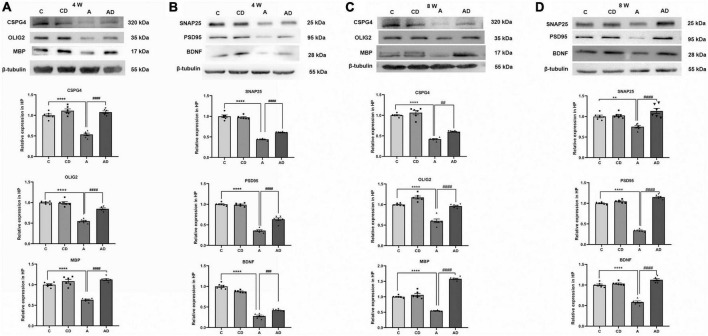
Effects of Disodium cromoglycate (DSCG) and general anesthesia (GA) on oligodendrocytes and synapses in hippocampus. **(A)** Representative western blot images and graphic presentation of CSPG4, oligodendrocyte transcription factor 2 (OLIG2) and myelin basic protein (MBP) protein abundance in 4 Weeks mice. **(B)** Representative western blot images and graphic presentation of synaptosomal associated protein 25 kDa (SNAP25), postsynaptic density protein 95 kDa (PSD95) and brain-derived neurotrophic factor (BDNF) protein abundance in 4 Weeks mice. **(C)** Representative western blot images and graphic presentation of CSPG4, OLIG2, and MBP protein abundance in 8 Weeks mice. **(D)** Representative western blot images and graphic presentation of SNAP25, PSD95, and BDNF protein abundance in 8 Weeks mice. Results are median ± S.E.M (*n* = 6) with the presence of individual animal data in the bar graphs. Results were analyzed by one-way analysis of variance (ANOVA) followed by the Tukey’s test. **P < 0.01, ****P < 0.0001 compared with C group. ^##^*P* < 0.01, ^###^*P* < 0.001, ^####^*P* < 0.0001 compared with A group.

Given the previous studies, overactivation of microglia and astrocytes leads to a persistent disruption of oligodendrocytes. We tested the expression of oligodendrocyte-associated protein in the hippocampus of each group of mice at different age stages (Figures 6A,C). Oligodendrocyte transcription factor 2 represented the differentiation level of oligodendrocytes, and MBP represented the mature oligodendrocytes after differentiation. Meanwhile, considering the relationship between oligodendrocytes and neuronal synapses (Liddelow et al., 2017), we further tested synapse-related proteins SNAP25, PSD95, and BDNF (Figures 6B,D). The results showed early GA exposure caused oligodendrocytes and synapses impairment, and DSCG rescued this effect.

## Discussion

The period from birth to adolescence is a golden stage for the rapid development of the nervous system. Early exposure to GA during the peak of brain development results in cognitive impairment (Zuo et al., 2022). Pathological mechanisms that regulate anesthesia-induced cognitive impairment have not been well characterized, even though neuroinflammation might provoke clinically cognitive dysfunction. Previous work showed that hippocampal inflammatory response might be the mechanism for cognitive impairment after isoflurane exposure (Yuan et al., 2019). In our study, long-term cognition was impaired by GA administration at PND 7 (the peak of synaptogenesis), and DSCG provided long-lasting protection until early adulthood.

Mast cells act as the first immune-related responders in the brain and can lead to the release of inflammatory factors through degranulation (Banafea et al., 2022). In this study, we demonstrated that MCs can promote the development of hippocampal inflammation after early GA exposure. For the pharmacological studies, we used stabilizer of MCs and showed the link between MCs degranulation and neuroinflammation. Tryptase is the major proteins in MCs and has been used as a marker for MCs activation and degranulation (Oliveira et al., 2013; Ocak et al., 2020). Our results showed that DSCG treatment decreased the expression of tryptase after early GA exposure. This might provide the direct mechanistic explanation for the protective effect of DSCG. In addition, our results revealed the unique role of MCs in the development of early GA exposure-related neuroinflammation. Our data showed that neuroinflammatory factor TNF-α and IL-1β were reduced in hippocampus after DSCG treatment.

Mast cells and microglia are found in close proximity to each other in the central nervous system, facilitating active communication (Zhang et al., 2016a). Our study found that degranulation of MCs activated microglia, and reactive astrocyte was induced by classically activated neuroinflammation microglia. Besides, overactivated microglia and astrocyte contributed to the impairment of maturation and differentiation of oligodendrocytes, and further impaired synaptic function. Neuroinflammation is the hub connecting factor and ultimately leads to cognitive impairment. Increasing evidence suggests that the initiation and propagation of neuroinflammation relies on the interactions between these cell types (Bi et al., 2013; Liddelow et al., 2017; Sandhu and Kulka, 2021). This study revealed long-lasting postnatal GA evidently induced cognitive impairment lasting into early adulthood by dysregulating the status of MCs, glia cells and synapses, and MCs stabilizer DSCG offered effectively protection.

Importantly, our study was first to reveal the following: (1) neonatal isoflurane exposure caused long-term cognitive impairment and lasting until early adulthood; (2) degranulation of MCs and subsequent dysregulation among MCs, glia cell subsets and synapses were involved in this process; (3) MCs were first responders to isoflurane-induced neuroinflammation; (4) MCs stabilizer DSCG can effectively inhibited MCs degranulation and further contribute to balance the dysregulation among MCs, glia cell subsets and synapses to ameliorate long-term cognitive impairment.

There are other limitations to this study. First, we did not explore sex-dependent differences in this study. Second, our study only performed in mice model *in vivo*. Further studies will be needed to explore sex-dependent differences in the contribution of MCs to the early GA exposure-induced long-term cognitive impairment, and combined with *in vitro* experiments to find the specific molecular mechanism involved to provide a more precise theoretical basis. Considering the clinical availability of mast cell stabilizer DSCG, our current study may provide the basis for future clinical studies to test mast cell stabilization for the treatment of GA exposure-induced long-term cognitive impairment.

## Conclusion

This study showed the first responder role of MCs in the promotion of the GA exposure-induced neuroinflammation and long-term cognitive impairment associated with neonatal mice. Mast cells stabilizer DSCG might serve as a potential therapeutic target for the treatment. Further mechanistic and clinical studies are needed to establish the contribution of DSCG in the process.

## Data availability statement

The raw data supporting the conclusions of this article will be made available by the authors, without undue reservation.

## Ethics statement

The animal study was reviewed and approved by the Institutional Animal Care and Use Committee (Approval No.: SYSU–IACUC–2022–000536) and the Laboratory Animal Ethics Committee of Sun Yat-sen University.

## Author contributions

FM and ZW conceived and designed the study and interpreted experiments. XZ and WW performed the experiments and prepared the initial draft of the manuscript. JJC revised the manuscript. ZZ, LL, JZ, LZ, and JWC participated and supervised the project. All authors read and approved the final submission.

## Abbreviations

GA: general anesthesia
PND: postnatal day
DSCG: disodium cromoglycate
MCs: mast cells
MCT: mast cell tryptase
IL: interleukin
TNF-α: tumor necrosis factor-alpha
C: sham controls
CD: sham + disodium cromoglycate treated
A: general anesthesia treated
AD: general anesthesia + disodium cromoglycate treated
PBS: phosphate buffer saline
CSPG4: chondroitin sulfate proteoglycan 4
OLIG2: oligodendrocyte transcription factor 2
MBP: myelin basic protein
SNAP25: synaptosomal associated protein 25 kDa
PSD95: postsynaptic density protein 95 kDa
BDNF: brain-derived neurotrophic factor
GFAP: glial fibrillary acidic protein
IBA1: ionized calcium binding adapter molecule 1
NOR: novel object recognition
CA1: cornus ammonis 1
PFA: paraformaldehyde
RT: room temperature.
